# On Poisoned Confectionary

**Published:** 1831

**Authors:** 


XXXII.

ON POISONED CONFECTIONARY. By W.B. O'SHAUGHNESSY, M.D.

WE fear that death is not confined to the POT alone; but that PASTRY performs its part in the tragedy of life. In France, where confectionary, sugar-plums, bonbons, and ten thousand species of sweetmeats, or rather beautified poisons, are still more in demand than here, the detection of deleterious matters in their colourings became an object of great importance, and the distinguished CHEVALLIER undertook the analytical research. This chemist detected in the Parisian confectionary various noxious substances, such as arsenic, copper, lead, mercury, gamboge, &c. and his report induced the French Government, who take cognizance of what enters as well as what issues from the mouth, sent forth an ordonnance prohibitory of pastry-cook poisoning in the good city of Paris, as well as elsewhere in the great nation. Dr. O'Shaughnessy, whose attention had, for some time previously, been directed to the subject, sat about investigating the colouring materials of the London pastry, and has recently brought the result of his researches before the public through the pages of the Lancet. From a separate paper with which we have been favoured by the author, we shall make a few extracts.

" On the subsequent day to that on which I perused the article just alluded to, I purchased, in company with my friend Dr. Green, at several shops, different specimens of coloured confectionary, and of colourless articles wrapped in stained paper. Of the coloured articles, the greater number (class 1) were sold expressly for eating, some (class 2) cast into small figures of cards, &c., were *apparently* rather intended for ornament, but were sold without restriction; and, lastly, some (class 3) were expressly designed for ornament alone. Of the first class I examined about thirty different kinds, and found the *reds* tinted as follows:

*Ten Specimens of Red Comfits, &c.*

1 Minium, or red oxide of lead.
2 Red sulphuret of mercury (vermilion.)
1 Mixture of both the former.
1 *Of a yellowish or orange tint*, chromate of lead, and a vegetable lake of lime.
2 Cochineal alone.
1 Cochineal, with a trace of *vermilion.*
2 Vegetable lakes of alumina and lime.
—
10

It is seen here, that of the ten specimens of comfits sold for eating expressly, six contained mineral poison; all these specimens, with one exception, were only coloured externally.

Of the *yellows*, class 1, seven specimens of different forms and tints. 4. *Gamboge*, coloured externally; 1. Coloured throughout a vegetable lake of lime; 1. *Coloured throughout*, oxide of lead, and traces of antimony, or Naples yellow. Six of the seven consequently contained deleterious substances.

Of the *greens*, class 1, several specimens, all were coloured by Prussian blue, and a vegetable yellow lake of alumina mixed with the sulphate of lime, except one specimen, of which I had only two comfits, and which gave me a mixture of copper and lime.

The *blues*, class 1, were chiefly Prussian blue, and contained no hurtful compound.

In the second class, or those *apparently* intended for ornament, but sold without restriction, and formed in all sorts of fantastic shapes, of eight forms of yellow, three contained chromate of lead; one Naples yellow; one massicot or yellow lead, and three vegetable lakes of alumina and lime. All these were coloured throughout, and contained moreover sugar, and the sulphate of lime or plaster of Paris.

The *reds* in this class were, of six specimens, three vegetable lakes of alumina or lime, one chromate of lead, with a red vegetable lake, two red lead.

The *greens* and *blues* were composed as I described in class 1.

In the *third* class the composition was precisely the same, and the proportion little different from class 2.

The papers were next examined, especially those used for enveloping the sugar drops called 'kisses.' Without exception the reds were coloured by the RED SULPHURET of MERCURY, the yellows by the CHROMATE of LEAD, and many of the greens by VERDEGRIS, or the carbonate of copper.*

With respect to the quantities of the poisonous substances, I had not leisure to submit the various products to the tedious process of delicate weighing. Moreover, it appears to me to be altogether unnecessary to take the trouble, as the mere presence of the minutest possible quantity of any such substance should not be allowed. In this opinion I entirely coincide with MM. Chevallier and Andral. It is perfectly unnecessary for me to occupy the pages of this Journal with any observations on the *nature* of the danger which thus threatens the junior branches of the community, and which indisputably exercises the most pernicious effects on their constitutions, I will merely remark that one concern in the city, from which I have obtained the greatest number of poisonous specimens, employs eleven men daily in the preparation of these articles, furnishes immense quantities of them to country confectioners, supplies many of the minor shops in the metropolis, and, if I am rightly informed, exports to our foreign possessions to a considerable amount. Extent of manufacture always implies extent of sale, and in this case the ratio of the consumption of course equals both. I cannot, therefore, be accused of exaggeration, when I assert that millions of children are thus daily dosed with metallic and vegetable poisons, in minute quantities it is true, but in quantities dependent on their amount on the caprice of a workman or a machine, and sufficient in the minutest degree to exercise their peculiar insidious effects, if taken as a practice from day to day. Neither are these effects chronic alone, for not long since an *acute* case of poisoning arising from the use of confectionary of this description occurred in the children of a highly respectable family in Southwark, and on analysis the comfits were found to contain minium, or the red oxide of lead."

Dr. O'S. next enters into the modes of analysing the different colours, especially the yellow, the red, the blue, and the green; but we cannot abridge his processes, and therefore shall give two of the most important analyses in the words of the author.

ANALYSIS OF THE YELLOW.

" [The yellows ordinarily met with, are coloured either by gamboge, massicot, Naples yellow, the chromate of lead, or vegetable lakes.]

The *yellows*, coloured by *gamboge* externally, when washed thus with distilled water, form an opaque yellow emulsion, which lets fall no deposit. By evaporating this emulsion to dryness, a little strong alcohol, added to the residuum, dissolves instantly the gamboge in a state of perfect purity. The alcoholic solution is then to be transferred to a test tube, and an equal quantity of distilled water added. The gamboge is now precipitated as a lively yellow; a drop or two of strong ammonia added, redissolves the gamboge, producing a blood-red solution, which again is precipitated pale yellow by the addition of nitric acid. This simple concatenation of experiments affords abundant proof of the presence of this substance. If the yellow colour proceeded from saffron, turmeric, or any other similar substance, it would form a *solution*, not an *emulsion*, with water. It would not be precipitated from its alcoholic solution by water, neither would it be precipitated from its ammoniacal solution by nitric acid. Two or three small comfits are amply suffi-

* "Sealed phials, containing specimens of the poisoned comfits, are left at THE LANCET Office for public inspection, in order to supersede the necessity of a description of their forms which could at best communicate a very faint idea of the pernicious kinds."

cient for the process, as it will detect the 100th part of a grain of gamboge. If great expedition be required, alcohol may be used at first to dissolve the colouring matter, and thus the tedious evaporation will be avoided.

If the *yellows*, when washed with water, and the comfits removed, let fall a yellow deposit, and leave the supernatant liquid colourless and transparent, the deposit is either the chromate of lead, Naples yellow *(oxide of lead and antimony)*, massicot (or the yellow oxide of lead), or, finally, a vegetable lake of alumina or lime. In most cases also, the precipitate contains sulphate of lime.

We can now readily gain a clue to which of these divisions it belongs, by the following simple system of trial tests, viz. by placing a minute portion of it, moistened with a small drop of distilled water on a thin slip of mica,* and holding it over the flame of a spirit lamp till it be heated to redness. If it be a vegetable lake of lime or alumina, it first chars, blackens, exhales smoke, and then leaves a brilliant, white, soft, earthy mass, entirely soluble in acetic acid when transferred to a watch crystal. A portion of this mass, if lime, stains moistened turmeric paper red; if alumina, has no such effect.

If, instead of charring and becoming white, the spot becomes red, and is surrounded with a little yellow circle, the colouring is massicot or yellow lead.

If, during this operation, dense white fumes are evolved, and leave a copious circle of the same colour on the mica, the substance, besides lead, *probably* contains antimony, and is, therefore, the Naples yellow.

If the colouring matter be chromate of lead, beautiful phenomena mark the action of heat, accomplished in the manner I describe; the substance on trial first darkens in colour, then shows a red surface, and by and by bright-green spots are seen mingled with the red. This contrast of colours becomes especially striking on the addition of a drop of water.

So far the experiments are trial tests, and may be performed in as short a time as it takes to read their description; our next object is to obtain unimpeachable evidence; this, in the case of the massicot, is acquired readily, by treating a grain or two of the yellow mass on a watch-glass with ten drops of nitric and six of muriatic acid, aiding the action by the momentary application of heat, white flakes of the chloride of lead now form and float on the acids; they are to be removed with a capillary tube, and transferred to a bit of firm charcoal, by directing the blowpipe flame on the cloride, it instantly fuses, and disengages globules of metallic lead, surrounded by concentric circles of red and yellow.

If the evolution of dense white fumes indicate the presence of antimony, the yellow matter should be treated, as just now described, with nitric and muriatic acid. The chloride of lead should be removed, and a few drops of distilled water poured on the residual fluid previously evaporated to dryness. If antimony be present in the smallest quantity, a white precipitate remains, which, if exposed to a current of sulphuretted hydrogen, expelled from a small bladder, furnished with a tube and stop cock, is converted into the orange-red sulphuret of antimony. The chloride of lead is then to be reduced by the blowpipe as before, and we have thus certain evidence of the presence of antimony and lead. Half a grain of the Naples yellow is sufficient for this chain of experiments.

If on the mica, green spots are mixed with the red, indicating chromate of lead, two or three grains of the remaining yellow matter should be fused for a quarter of an hour on a slip of mica, with an equal quantity of nitrate of potash, chromate of potash is thus formed, the green spots disappear, and red flocks of minium, or the red oxide of lead, are seen in the fused nitre. The fusion should now be discontinued, and the cooled mass dissolved in distilled water on a watch-glass; the solu-

* Mica can be procured at the mineralogists' shops at a very cheap rate, it is of immense service in minute analyses of this kind. (See LANCET, No. 396.)

tion should then be separated from the red oxide by a capillary tube, and transferred to another crystal. Nitrate or acetate of lead will now occasion in it a precipitate of the yellow chromate of lead. The red flocks are to be heated with nitro-muriatic acid, and the resulting chloride of lead reduced on charcoal to the metallic state.

The protracted fusion of the nitrate of potash may be easily accomplished, by placing a slip of mica four inches long and two broad, over the mouth of a wine glass, so that it can be balanced by a shilling placed on it over the glass; about two inches thus project, beneath which is placed a spirit lamp, and the fusion may be kept up an unlimited length of time. To those unprovided with costly apparatus, this simple substitute may prove, as it has to me, of considerable value.

ANALYSIS OF THE RED.

The red, on being washed or boiled with water, either form a coloured transparent solution, which affords no deposit, and filters through paper, or is coloured and affords a deposit, or affords a dense deposit, and leaves the fluid transparent and colourless.

In the first case, the solution is entirely decolorized by chlorine, or by dropping in a particle of the chloride of lime. If a second portion of it be changed to orange-yellow by sulphuric acid, and a third assume a violet with ammonia, and if no black colour is produced by adding the sulphate of iron, it may be concluded to be a solution of *cochineal.*

If a deposit takes place, which, when dried and heated on mica, chars, blackens, and finally becomes white, and the residuum soluble in acetic acid, it is a vegetable lake of alumina or lime, probably carmine.

If the deposit be a bright-red colour, and subside rapidly, it is probably either the red oxide of lead, or the sulphuret of mercury; in either case heat on mica; if the former, the colour remains unchanged, and the substance is permanent at a red heat. If the latter, it is darkened on the slightest application of heat; if then removed, it regains on cooling a brilliant vermilion colour. This alternate blackening and reddening may be repeated *ad libitum*, till it is finally volatilised, leaving no trace behind.

So far, as in the analysis of the yellows, the experiments are but trial tests. The reduction of the respective metals is next to be accomplished. This is easily effected, by boiling a few particles of the colouring matter in nitro-muriatic acid. If the trial test has pointed out the sulphuret of mercury, on evaporating nearly to dryness, brilliant crystals form on the watch-glass, these should be redissolved in a few drops of distilled water, acidulated with nitric acid, and a gold ring with a bit of thin iron wire introduced. If the colouring matter contains the one-thousandth part of a grain of mercury, it will be thus deposited on the gold as a white amalgam.

If the red colour was permanent on the mica, the colouring matter is to be heated with nitro-muriatic acid as before described, and reduced on charcoal to the state of metallic lead."

The author, urged by a sense of public duty, laid the present subject before the Home Secretary; but how far his representations may lead to any legislative measures, is uncertain — or rather, we may safely say, hopeless. The members of Government, in this country, are too deeply immersed in political contests, to pay much heed to the public health — a subject which has always been neglected by our legislature, even upon much more important points than confectionary. It appears that the only enactments respecting the SALUS PUBLICA, which Coleridge thought worthy of insertion in the latest edition of Blackstone's Commentaries, are those which enforce the observance of quarantine, which prohibit the *adulteration of wine*, the sale of unwholesome meat —*or meat bought of a Jew!* It is surely a monstrous shame that the poor Hebrew should not be allowed to sell his pork, seeing that he can make no use of it in his own kitchen!

Dr. O'Shaughnessy is evidently a clever chemist, and his industry appears equal to his talent in this department of human knowledge.

